# Scar endometriosis: a mimic of acute abdominal emergencies

**DOI:** 10.1259/bjrcr.20170019

**Published:** 2017-04-01

**Authors:** Dylan Paul Roi, Joel Lewis Schamroth, Lubna Khalid, Ajay Sahu

**Affiliations:** ^1^Radiology department, Imperial College healthcare NHS trust, London, UK; ^2^Academic department, Royal Brompton and Harefield NHS, London, UK; ^3^Radiology department, North West London Healthcare NHS trust, Harrow, UK

## Abstract

Scar endometriosis can be a great mimic of common surgical and gynaecological conditions in reproductive age females. The correct preoperative diagnosis is only achieved in a relatively low number of patients (20–50%). This uncommon condition presents diagnostic challenges and requires surgical excision in most cases. Ultrasound represents a useful tool to triage and direct management as well as further investigations. We present a case of scar endometriosis in a young female with a previous history of caesarean section.

## Clinical presentation

A 29-year-old female presented to accident and emergency at our local hospital with 2 days of acute abdominal pain. She reported no pyrexia, diarrhoea or vomiting. She had complained of cyclical pain over the preceding few months and had a past history of two  caesarean sections and endometriosis. She took no regular medications, and had received courses of non-steroidal anti-inflammatory analgesia and co-dydramol.

Her admission blood tests were normal including a negative serum beta human chorionic gonadotropin. Serum CA-125 measurements were not taken in this case. On examination, she was exquisitely tender in the midline suprapubically with no rebound tenderness.

## Differential diagnosis

The differential list includes non-specific gastrointestinal causes, appendicitis and hernias. Gynaecological aetiologies include ovarian pathology or ectopic pregnancy. Rarely, abdominal wall tumours such as desmoids, lipomas or sarcomas can also present in a similar manner. Urological causes such as urinary tract infections or a ureteric stone should be considered. In the first months following surgery, suture granulomas or haematomas can be seen. However, a cyclical nature to the pain in relation to a previous scar is strongly suggestive of scar endometriosis.

## Investigations/imaging findings

**Figure 1.**
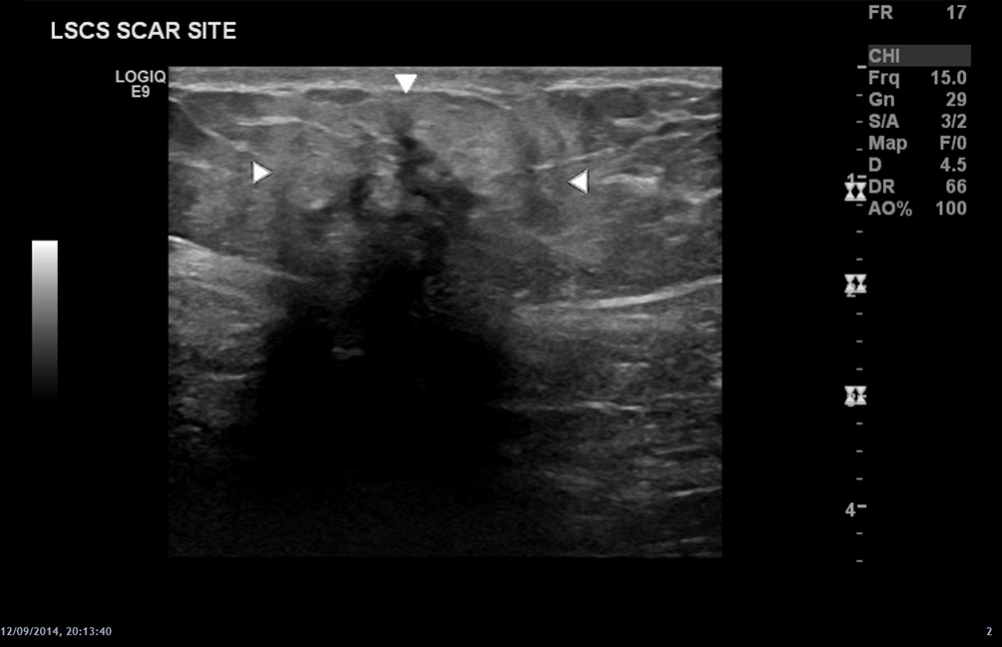
Hypoechoic, irregular lesion at the site of caesarean scar suggestive of scar endometriosis.

**Figure 2.**
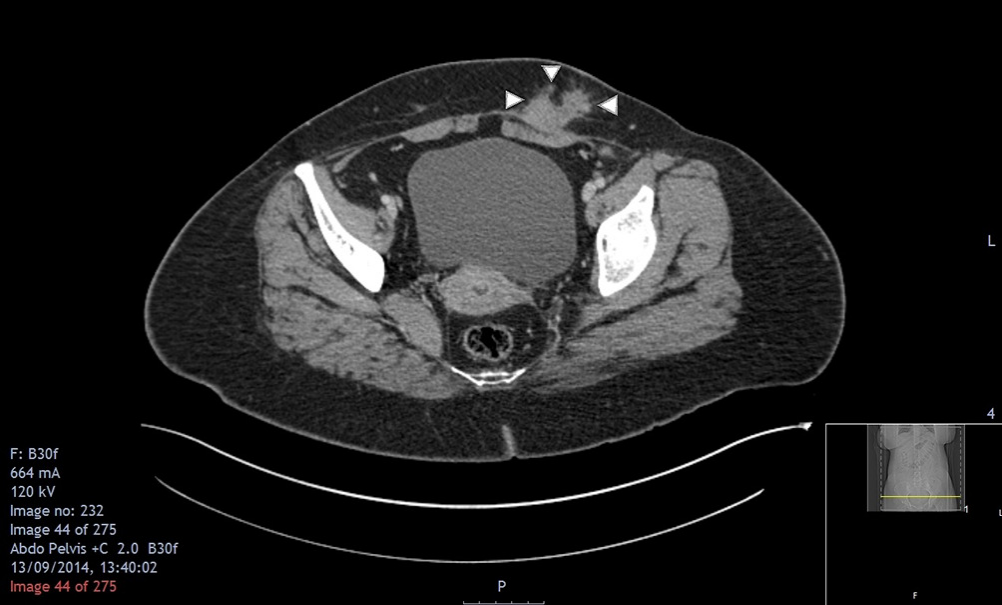
Axial contrasted CT image showing a well-defined soft tissue nodule with postcontrast enhancement in the soft tissue, and streakiness in the surrounding tissue, extending anteriorly from the rectus muscle.

The first line investigation for this patient was ultrasound. A full abdominal ultrasound as well as a transabdominal pelvic ultrasound were carried out. Sonographic appearances of the abdominal and pelvic ultrasound were normal other than the lesion seen in Figure 1. An irregular hypoechoic focus underlying the caesarean section scar was demonstrated. This was further assessed with CT (Figure 2) revealing a well-defined lesion of increased density post contrast administration (CT protocol: abdomen and pelvis with portal phase intravenous contrast (at 70 s), range: from diaphragm to below symphysis pubis; with 2 mm reconstructions).

## Treatment

The findings were discussed at the local multidisciplinary team (MDT) meeting and treatment options were presented to the patient. She elected to undergo local excision. This was an uncomplicated procedure. The soft tissue specimen underlying the abdominal scar measured 40 × 30 × 25 mm. It was described as “fibrofatty tissue with secretory endometrial glands and surrounding stroma, diagnostic of endometriosis.”

## Outcome and follow-up

The patient was well when followed up postoperatively. She remained symptom free when contacted a year later.

## Discussion

Ectopic endometrial tissue is responsive to the same hormonal cycle as the uterine endometrium. This process outside of the uterus leads to the well-defined constellation of symptoms that characterizes endometriosis, infertility, dysmenorrhoea, pelvic/back pain, menorrhagia, dyspareunia and pain on defecation.^1^ Scar endometriosis occurs when endometrial tissue develops at the site of scar tissue following surgical abdominal procedures.^2,3^ This is associated with caesarean section, hysterectomy and more rarely with non-gynaecological abdominal surgery. The incidence of scar endometriosis following it is estimated to be between 0.03 and 1.7%^2,4–7^ with a mean patient age at presentation of 31.4 years.^8^ The mean interval from caesarean surgery to time of presentation is typically 3–4 years; however, this is highly variable.^4,8^ There may also be a period of latency between mass formation and onset of pain.^9^ Interestingly, although our case had a past history of endometriosis, which may have assisted in diagnosis, overall a low proportion of affected patients present with concurrent pelvic endometriosis.^8^

Pain and the presence of a mass at the site of Pfannensteil caesarean section scar were the most common features of scar endometriosis.^8^ The prevalence of cyclical pain in scar endometriosis has been estimated at between 20% and 57%.^8,10^ However, when present, a painful mass at a caesarean scar site where pain fluctuates with the menstrual cycle is pathognomonic for scar endometriosis.

The nodules are often found deep in the subcutaneous plane in contact with muscularis fascia, often infiltrating the rectus sheath or rectus abdominis.^11^ The average lesion diameter seen on ultrasound is 28.1 mm (n = 12, range 7–50 mm),^11^ which matches the mean size of 27 mm in surgically excised lesions (pooled data from 13 studies reviewed).^8^ As the lesion grows it can lose its round or oval shape and undergo cystic degeneration.^11^

They usually feature an inhomogeneous hypoechoic texture,^9,12^ but may show scattered high echoes.^11^ The can appear lobulated^9^ or septated,^2^ with uniform margins,^13^ which may become more irregular with increasing size.^11^ Mild vascularity is frequently seen on Doppler.^14^ It may depend on size, with small lesions showing no vascularity, in contrast to large lesions having intralesional vascularity. A single arterial vascular pedicle is often seen supplying these lesions.^14^ Localized tenderness can be elicited with ultrasound as was seen in the present case. Ultrasound can also be used to obtain targeted tissue biopsies.

Scar endometriomas can be assessed with other modalities. CT typically shows a solid and well-circumscribed mass^12^ (Figure 2). MRI is preferred as it is good at characterizing small lesions by distinguishing the haemorrhagic signal that can be a feature of endometriotic lesions. It is also useful to differentiate soft tissue layers in the abdominal wall.^15^ MRI is more specific and sensitive but may not always be readily available. Definite diagnosis requires histopathological examination of a sample of the lesion,^9^ and discussion in Gynae MDT as a soft tissue sarcoma cannot be entirely excluded on imaging features.

Once confirmed on histopathology, conservative management options include oral contraceptives, progestins such as medroxyprogesterone acetate and gonadotropin-releasing hormone agonists, although these are usually not sufficient. Most cases require surgical excision.^13^ Post resection recurrence is reported in 4.3% of cases.^8^ Management choices should account for the potential for rare malignant transformation of endometriomas (0.3–1%), such as adenocarcinoma, sarcoma or clear cell carcinoma in order of prevalence.^16^ An MDT meeting approach can facilitate management and decision making.

## Learning points

1. Scar endometrioma should be considered in females of reproductive age with a previous history of caesarean section presenting with cyclical pain. Radiologists and surgeons need to be aware of this entity and consider it in their differential list.
2. The diagnosis of scar endometriosis requires a high degree of suspicion based on a thorough surgical and gynaecological history and clinical examination.
3. Ultrasound can be used to narrow down the list of diagnoses and guide management, and should be the first line of investigation in reproductive age females because it does not involve radiation and because of the ease of availability.
4. Endometrioma can mimic malignant lesions and should therefore warrant a biopsy if not surgically excised.

## Consent

Written informed consent for the case to be published (including images, case history and data) was obtained from the patient(s) for publication of this case report, including accompanying images.
